# Association of a bitter taste receptor mutation with Balkan Endemic Nephropathy (BEN)

**DOI:** 10.1186/1471-2350-13-96

**Published:** 2012-10-11

**Authors:** Stephen P Wooding, Srebrena Atanasova, Howard C Gunn, Rada Staneva, Invanka Dimova, Draga Toncheva

**Affiliations:** 1 McDermott Center for Human Growth and Development, University of Texas Southwestern Medical Center, Dallas, TX 75244, USA; 2 Department of Clinical Chemistry, Georg-August University, Robert-Koch-Strasse 40, 37075, Göttingen, Germany; 3 Department of Medical Genetics, Medical University of Sofia, Sofia, 1431, Bulgaria

## Abstract

**Background:**

Balkan Endemic Nephropathy (BEN) is late-onset kidney disease thought to arise from chronic exposure to aristolochic acid, a phytotoxin that contaminates wheat supplies in rural areas of Eastern Europe. It has recently been demonstrated that humans are capable of perceiving aristolochic acid at concentrations below 40 nM as the result of high-affinity interactions with the TAS2R43 bitter taste receptor. Further, TAS2R43 harbors high-frequency loss-of-function mutations resulting in 50-fold variability in perception. This suggests that genetic variation in TAS2R43 might affect susceptibility to BEN, with individuals carrying functional forms of the receptor being protected by an ability to detect tainted foods.

**Methods:**

To determine whether genetic variation in TAS2R43 predicts BEN susceptibility, we examined genotype-phenotype associations in a case–control study. A cohort of 88 affected and 99 control subjects from western Bulgaria were genotyped with respect to two key missense variants and a polymorphic whole-gene deletion of TAS2R43 (W35S, H212R, and wt/Δ), which are known to affect taste sensitivity to aristolochic acid. Tests for association between haplotypes and BEN status were then performed.

**Results:**

Three major TAS2R43 haplotypes observed in previous studies (TAS2R43-W35/H212, -S35/R212 and –Δ) were present at high frequencies (0.17, 0.36, and 0.47 respectively) in our sample, and a significant association between genotype and BEN status was present (P = 0.020; odds ratio 1.18). However, contrary to expectation, BEN was positively associated with TAS2R43-W35/H212, a highly responsive allele previously shown to confer elevated bitter sensitivity to aristolochic acid, which should drive aversion but might also affect absorption, altering toxin activation.

**Conclusions:**

Our findings are at strong odds with the prediction that carriers of functional alleles of TAS2R43 are protected from BEN by an ability to detect and avoid aristolochic acid exposure. Evidence for a positive association between high-sensitivity alleles and BEN status suggests instead that possession of toxin-responsive receptor variants may paradoxically increase vulnerability, possibly by shifting attractive responses associated with low-intensity bitter sensations. The broad-spectrum tuning of the ~25-member TAS2R family as a whole toward xenobiotics points to a potentially far-reaching relevance of bitter responses to exposure-related disease in both individuals and populations.

## Background

**Figure 1:**
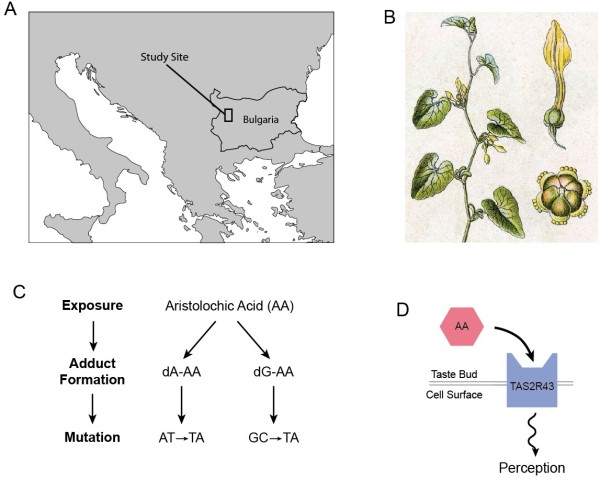
**Epidemiological and biological background. A **Location of study population. **B ***Aristolochia clematitis *plant, flower, and seeds [7]. **C **Pathway from aristolochic acid exposure to mutagenesis (modified from [8] Figure 6.) **D **Mechanism of taste response to aristolochic acid.

Balkan endemic nephropathy (BEN) is a late-onset kidney disease occurring at high frequency in localized areas of Eastern Europe [1-4]. Following asymptomatic early stages, affected individuals suffer progressive development of anemia, proteinuria, kidney atrophy, high rates of urothelial cancer, and eventually death [1]. BEN’s geographical distribution, which is restricted to rural agricultural areas, along with non-familial aspects of inheritance, suggested as early as the 1950s that the disease is caused by an environmental agent [5]. Recent findings on BEN’s molecular pathology, including an enrichment of aristoclactam-DNA adducts in tumor tissues, have demonstrated that it arises from exposure to aristolochic acid, a potent nephrotoxin produced by plants in the genus *Aristolochia*[6] (Figure 1). This compound enters food supplies when the seeds of *Aristolochia clematitis*, a weed common in wheat fields, are unintentionally harvested along with the crop [5].

Toxicity is a ubiquitous mechanism of defense in plants, which utilize a constellation of secondary metabolites to deter herbivores, including compounds targeted at fundamental biological processes such as embryonic development (e.g., cyclopamine), hormonal homeostasis (e.g., goitrin), and protein synthesis (e.g., ricin). Numerous plant toxins are perceived as bitter, even at low concentrations, and it has long been hypothesized that bitter sensitivity serves as “toxin detector” that enables animals to identify and avoid dangerous foods [9]. The importance of this role declined in humans with the advent of technological innovations such as domestication and industrial processing. However, bitter taste retains health relevance as a mediator of toxic exposures via contamination and spoilage [10,11]. Further, the range of compounds perceived as bitter extends beyond natural sources to include many pharmaceuticals and synthetic agents, suggesting that bitter responses may mediate clinically-significant exposures in man-made environments [12,13]. Nonetheless, specific instances of taste-toxin interaction, which are likely to be informative about both the underlying mechanisms and epidemiological consequences of exposure, remain poorly documented.

Isolation of aristolochic acid from *A. clematitis* confirmed more than a century ago that it is abundant in the plant and, like many phytotoxins, possesses aversive taste characteristics [14]. Whether bitter perception plays a role in regulating aristolochic acid exposure has remained unknown; however, Pronin et al. [15] recently demonstrated that humans are capable of perceiving aristolochic acid at concentrations lower than any other natural compound identified to date, with some subjects reliably detecting it at just 40 nanomolar. Further, individuals vary more than 50-fold in threshold sensitivity as the result of high-frequency loss-of-function mutations in TAS2R43, a bitter taste receptor exhibiting high affinity for aristolochic acid [12,15,16]. These findings suggest that variation in TAS2R43 could be an important mediator of BEN susceptibility, with high-function alleles conferring protection by recognizing tainted foods. If so, genotype could be a useful tool for predicting clinically-significant vulnerability in individuals or populations. To examine this relationship, we performed a case–control study testing for association between mutational variants in TAS2R43 and disease status in a BEN-affected population.

## Methods

### Subjects

Subjects were recruited from the Vraza-Sofia region of Bulgaria as part of an ongoing epidemiological study [3,17,18]. The case group was composed of 88 non-related BEN patients (20% male and 80% female; mean age 63 ± 14 years). Diagnosis was made based on several consensus criteria: place of birth and residence in an endemic village, family history of endemic nephritis, anemia, specific color of face and palms, absence of arterial hypertension, proteinuria, low specific gravity of the urine, retention of nitrous compounds in the blood (urea >8.3 mMol/L, creatinine >133 μMol/L), and shrunken kidneys. Seven were diagnosed with renal cancer and four with bladder cancer. The control group was composed of 99 non-related healthy volunteers without family history of renal disease or cancer, and matched with cases for sex (25% male and 75% female) and age (58 ± 10 years). Written informed consent was obtained from all participants under human subject protocols in compliance with the Helsinki Declaration, which were approved by the home institution of DT, the Medical University of Sofia.

### Genotyping

In a previous study of genetic effects on aristolochic acid perception, Pronin et al. [15] demonstrated that two tightly linked single-nucleotide polymorphisms (SNPs) in *TAS2R43*, C104G/S35W and A635G/H212R (rs68157013 and rs71443637), define functionally divergent alleles associated with threshold detection in subjects. While TAS2R43-W35/H212 exhibits strong response in heterologous expression assays (EC_50_ = 8 nM) and is associated with high taste sensitivity, TAS2R43-S35/R212 exhibits weaker response (EC_50_ = 73 nM) and is associated with low sensitivity. Some evidence suggests that a second bitter taste receptor, TAS2R44, may contribute to aristolochic acid perception; however, functional assays indicate that TAS2R44 is substantially less responsive than is TAS2R43, and variation in TAS2R44 is not associated with taste sensitivity [15]. Therefore, only the TAS2R43-W35/H212 and -S35/R212 SNPs were considered as candidates in our study. To determine subject genotypes with respect to these two variants, we utilized allelic discrimination assays, which were carried out using an Applied Biosystems Incorporated (ABI) HT7900 Real-Time PCR system with TaqMan probes and reagents purchased from ABI. Phenotypic measures of taste response to aristolochic acid, which were obtained from healthy subjects by Pronin et al. [15], were not obtained in our subjects owing to the dangers of further exposing an at-risk population.

### Copy-number assays

In addition to the C104G/S35W and A635G/H212R mutations, *TAS2R43* is known to harbor a high-frequency whole-gene deletion allele (*TAS2R43*-Δ) in European populations [15,19]. To characterize genotypes with respect to the deletion, we performed copy-number assays using methods and reagents developed by Roudnitzky et al. [19]. In brief, we utilized a real-time quantitative PCR incorporating two primer sets and two fluorescently labeled TaqMan® MGB probes to determine whether a given subject harbored 0 copies of *TAS2R43* (*i.e.*, is homozygous for the deletion), 1 copy (heterozygous for the deletion), or 2 copies (does not carry the deletion). These data were then combined with the SNP data using the method of Roudnitzky et al. [19] to produce final genotypes, which were characterized by three alleles, C, G, and Δ at position 104 and A, G, Δ at position 635.

### Association tests

Tests for association between *TAS2R43* mutations and BEN were performed using the R statistical package [20]. To take into account co-dominance effects identified in previous studies of association, which have found that individuals heterozygous with respect to functionally divergent TAS2R variants display intermediate phenotypes, subjects were coded as carrying 0, 1, or 2 copies of the functionally responsive TAS2R43-W35/H212 allele. Fisher’s Exact tests were then performed on a contingency table of genotype with respect to functional alleles and case/control status. The results of these analyses were confirmed using linear regression and *χ*^*2*^ tests.

## Results and discussion

Previous studies have demonstrated that the TAS2R43 bitter taste receptor is a key mediator of aristolochic acid perception. Functional forms of the receptor exhibit detectable responses at concentrations of ~10 nanomolar in heterologous expression assays, and some individuals reliably perceive aristolochic acid at concentrations as low as 40 nanomolar [15,16]. It has also been demonstrated that ability to perceive aristolochic acid is influenced by common functional mutations in TAS2R43 including two tightly linked amino acid variants, C104G/S35W and A635G/H212R (rs68157013 and rs71443637), and a whole-gene deletion, TAS2R43-Δ. While TAS2R43-W35/H212 exhibits strong response to aristolochic acid in heterologous expression assays and is associated with low threshold detection (*i.e.*, high taste sensitivity), TAS2R43-S35/R212 and TAS2R43-Δ exhibit weak or no response and are associated with high threshold (*i.e.*, low sensitivity). These findings have suggested that variation in TAS2R43 might affect susceptibility to BEN, with TAS2R43-W35/H212 conferring protection by signaling aristolochic acid contamination in food.

**Figure 2:**
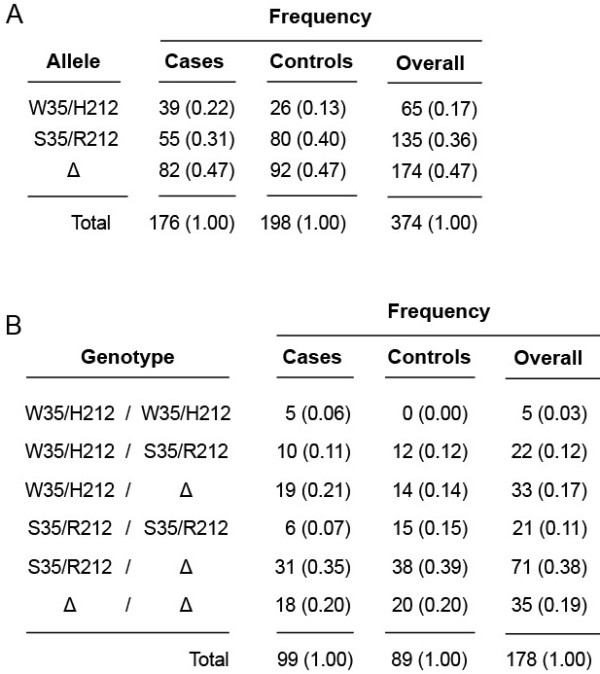
**Genetic data. A**Occurrence of TAS2R43-W35/H212, -S35/R212 and Δ alleles in subjects. Counts are given, with frequencies shown in parentheses. **B **Genotype frequencies in subjects.

SNP genotyping and copy-number determination revealed that the three major functional variants of TAS2R43 (TAS2R43-W35/H212, -S35/R212 and –Δ) were all common in our sample, with frequencies of 0.17, 0.36, and 0.47 respectively. No recombinant variants of TAS2R43-W35/H212 and -S35/R212 (e.g., -W35/R212) were observed, such that W35S and H212R were in perfect linkage disequilibrium (LD) (*r*^*2*^ = 1). These patterns are consistent with those reported by Roudnitzky et al. [19], who observed frequencies of 0.15, 0.35, and 0.50 in a similar panel of European subjects, with tight linkage (*r*^*2*^ = 1) between W35S and H212R. Overall, 2.7% (n = 5) of subjects were homozygous for the TAS2R43-W35/H212 allele, 29.4% (n = 55) were heterozygous, and 67.9% (n = 127) were non-carriers (Figure 2). Again, these values are comparable to those observed by Roudnitzky et al. [19] (3.3%, 23.3%, and 73.3%). Thus, levels and patterns of variation in our sample appear to be typical for European populations. However, they are not necessarily typical for other populations. Pilot data from the 1000 Genomes Project (1000GP), a consortium-based effort to sequence more than 2000 complete human genomes with worldwide representation, indicate that TAS2R43-S35/R212 is present at frequencies <3% in Asian populations [21].

**Figure 3:**
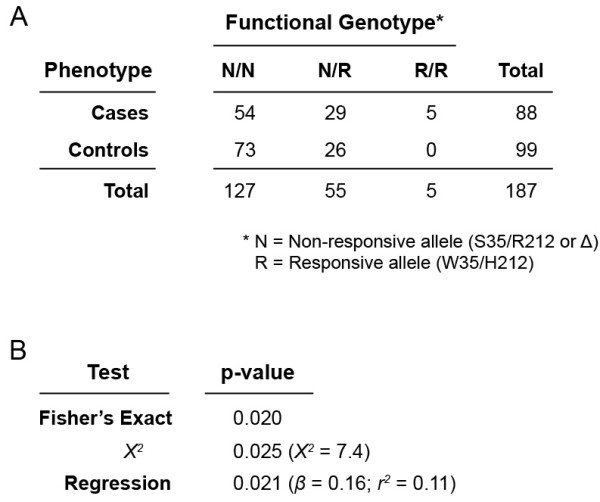
**Association data and tests. A **Frequencies of genotypes with respect to functionally divergent allele categories N (Non-responsive to aristolochic acid; W35/H212) and R (Responsive to aristolochic acid; S35/R212 and Δ). **B **Results of regression, Fisher’s Exact, and *Χ*^*2*^ tests for association.

Association tests detected a significant relationship between TAS2R43-W35/H212 carrier status and BEN; however, counter to expectation, the correlation was positive, not negative as would be predicted if perception of aristolochic acid leads to avoidance (Figure 3). Fisher’s exact test yielded a *P*-value of 0.020, with the case population being enriched with TAS2R43-W35/H212. Tests using linear regression indicated the presence of an effect with an estimated odds ratio of 1.18 (*P =* 0.025). Similar *P*-values were obtained using linear regression (*P* = 0.021; *β* = 0.16; *r*^2^ = 0.11) and *χ*^*2*^ tests (*χ*^*2*^ = 0.74; *P =* 0.025). Thus, significance levels were not sensitive to the analytical model. These findings are at odds with the prediction that aristolochic acid-sensitive TAS2R43 alleles provide an effective safeguard against overexposure, and raise the paradoxical possibility that they instead confer susceptibility.

Evidence that aristolochic acid-sensitive alleles confer increased susceptibility to BEN is striking and counterintuitive. Bitter perception has long been hypothesized to provide protection against toxins in the environment, particularly plant toxins, by enabling detection and avoidance [22]. This hypothesis is supported by much circumstantial evidence: bitter tastes are generally aversive, plant-derived toxins are over-represented among known bitter tastants, bitter taste receptors respond to many well-known toxins, and plants containing these toxins are perceived as bitter [9,12]. Thus, the clear prediction under the toxin-detector hypothesis is that TAS2R43-W35/H212, which has been shown to be associated with low taste thresholds for aristolochic acid, should provide a warning signal providing protection against BEN or, if aristolochic acid concentrations are too low to perceive, have no effect on susceptibility. Our finding that TAS2R43-W35 is positively, rather than negatively, associated with BEN is strongly at odds with this prediction.

One explanation for our findings could be that, contrary to expectations, the bitter taste of aristolochic acid is attractive to those who perceive it, rendering them susceptible to disease-causing levels of exposure. This explanation conflicts with predictions under the toxin-detector hypothesis; however, it is consistent with psychophysical studies, which have revealed that while intense bitter sensations are universally aversive, mild sensations can evoke positive responses [23]. Positive aspects of bitterness are evident in everyday experience; many foods, such as beer and salad greens have bitter, yet overall pleasurable, taste characteristics. This explanation is consistent with evidence that BEN arises as the result of chronic low-level exposures, which might be perceived as pleasantly bitter, as opposed to acute high-level exposures, which would likely be aversive.

A second, more speculative, explanation for our results is suggested by reports that bitter taste receptors and other major components of the taste transduction pathway are expressed in the mammalian gut, as well as on the tongue [24]. In mice and rats, Tas2R transcripts are found in gastric and duodenal tissues, and cells derived from intestinal tissue show dosage-dependent responses to bitter tastants [25]. The native role of TAS2R-mediated signaling in the gut remains unclear, but signals initiated by these receptors can induce the release of signaling peptides from enteroendocrine cells [25]. Further, some evidence suggests that post-absorptive metabolic activation of aristolochic acid is crucial to its toxicity [26]. If the response of gut-expressed *TAS2R43* to aristolochic acid enhances either absorption or metabolism, then genetic variation in *TAS2R43* might shape susceptibility to BEN.

BEN’s distinctive pathology is not unique. Similar patterns of kidney damage and increased rates of urothelial cancer have emerged in subjects overexposed to herbal supplements containing aristolochic acid [2]. Based on the etiological similarities of these diseases, it has been suggested that they be aggregated under an umbrella term, aristolochic acid nephropathy (AAN). To date, efforts to find genetic variants accounting for variable susceptibility to AAN have been relatively unsuccessful. For instance, variants in several genes (*NQO1*, *GSTT1*, *GSTM1*, *NAT1*, and *NAT2*) found previously to be associated with occupationally-induced renal and bladder cancers were not associated with AAN diagnosis [17]. Our findings recommend that variation in *TAS2R43* be considered a candidate factor in overall AAN susceptibility. While taste sensitivity alone seems unlikely to account for variable exposure aristolochic acid via nutritional supplements, which are often encapsulated to disguise taste, TAS2R43 variation might exert effects through activity in the gut.

## Conclusions

From clinical and epidemiological perspectives, our findings have two primary implications. First, evidence that mutational polymorphism TAS2R43 significantly affects susceptibility to BEN points to a potentially broad role for toxin responses in personal health risks. Most of the ~25 known TAS2Rs in humans exhibit responses to both phytotoxins and synthetic molecules, and many harbor extensive genetic diversity [12,27]. Thus, normal variation in taste or other TAS2R-mediated traits could be a appreciable contributing factor in individual exposure. Indications that susceptibility to BEN arises, in part, from inherited mutations also has epidemiological implications. In particular, it is well established that mutations in *TAS2R* genes vary substantially in frequency among populations [27-29]. For this reason, variation in TAS2R-mediated traits, and therefore susceptibility, could differ among groups. This possibility is exemplified by early results from the 1000GP, which indicate that while TAS2R43-S35/R212 is present at frequencies >30% in Europeans, it accounts for <3% of alleles in Asian groups. We anticipate that advances in large-scale *in vitro* deorphanization of TAS2Rs, which are capable of rapidly identifying candidate receptor-agonist interactions, will be a valuable tool in identifying and determining the significance of these patterns.

## Competing interests

The authors declare that they have no competing interests.

## Authors’ contributions

SPW and DT conceived and supervised all research activities. HCG performed laboratory genetic analysis. SA, RS, and ID performed laboratory work and sample preparation. All authors read and approved the final manuscript.

## Pre-publication history

The pre-publication history for this paper can be accessed here:

http://www.biomedcentral.com/1471-2350/13/96/prepub
